# Human blood vessel microbiota in healthy adults based on common femoral arteries of brain-dead multi-organ donors

**DOI:** 10.3389/fcimb.2022.1056319

**Published:** 2022-11-30

**Authors:** László Hidi, Gergely Imre Kovács, Dóra Szabó, Nóra Makra, Kinga Pénzes, János Juhász, Péter Sótonyi, Eszter Ostorházi

**Affiliations:** ^1^ Department of Vascular and Endovascular Surgery, Heart and Vascular Center, Semmelweis University, Budapest, Hungary; ^2^ Institute of Medical Microbiology, Semmelweis University, Budapest, Hungary; ^3^ Faculty of Information Technology and Bionics, Pázmány Péter Catholic University, Budapest, Hungary

**Keywords:** vascular, blood vessel, microbiota, allograft, 16S rRNA

## Abstract

Discovery of human microbiota is fundamentally changing our perceptions of certain diseases and their treatments. However little is known about the human blood vessel microbiota, it may have important effects on vascular pathological lesions and vascular homograft failure. In our prospective survey study fourteen femoral arteries, harvested from donors in multi-organ donations, were examined using the V3-V4 region 16S rRNA sequencing method. The most abundant phyla in the human vascular microbiota were *Proteobacteria*, *Firmicutes* and *Actinobacteria*. At the genus level, the most abundant taxa were *Staphylococcus*, *Corynebacterium*, *Pseudomonas*, *Bacillus*, *Acinetobacter* and *Propionibacterium*. Of the bacterial taxa that have an indirect effect on the development of atherosclerosis, we found *Porphyromonas gingivalis*, *Prevotella nigrescens* and *Enterobacteriaceae* spp. with different abundances in our samples. Of the bacteria that are more common in the intestinal flora of healthy than of atherosclerosis patients, *Roseburia* and *Ruminococcus* occurred in the majority of samples. The human arterial wall has a unique microbiota that is significantly different in composition from that of other areas of the body. Our present study provides a basis for ensuing research that investigates the direct role of the microbiota in vascular wall abnormalities and the success of vascular allograft transplantations.

## Introduction

Traditionally, most of the internal organs and blood are considered sterile. However, recent evidence has challenged this dogma by molecular-based detection of microorganisms in different tissues of healthy and non-healthy individuals (Paisse et al., 2016; Mansour et al., 2020; Molina et al., 2021; Sookoian and Pirola, 2021). Moreover, some authors could identify biologically relevant living microorganisms in these “sterile” regions (Clifford and Hoffman, 2015; Potgieter et al., 2015; Moreno et al., 2016). These insights, and the discovery of human microbiota, can fundamentally change our perceptions of certain diseases and their treatments.

The vascular system has been also considered a sterile environment. However, microorganisms can be detected in some vascular diseases in the arterial wall (aneurysm, atherosclerosis, vasculitis) (Blanchard et al., 1993; Kozarov et al., 2005; Rosenfeld and Campbell, 2011; Clifford and Hoffman, 2015). While microbiota research of gut and other traditionally non-sterile areas is in an advanced stage, little is known about the vascular wall tissue microbiota. We have collected a growing pool of knowledge about the cardiovascular effects of intestinal microbiota’s metabolites (Jie et al., 2017; Liu et al., 2019; Verhaar et al., 2020; Jin et al., 2021). However, little information is available on the local vascular microbiota and its possible role in homeostasis or pathological processes. It is often argued that detected bacterial DNA in sterile areas of the human body is just a residual fragment of dead bacteria. However, biologically active metabolites and antigens derived from the same bacteria also present may play important roles in various pathological or autoimmune processes. In addition, the relationship between outcome of liver or renal transplantation and the gut, oral or urinary microbiota has been repeatedly reported (Campbell et al., 2020; Dery et al., 2020). At this point, we have no information about relationship of vascular allografts and any microbiota, although vascular graft failures are common and have no clear explanation (Chakfe et al., 2020). On one hand, knowledge about the human vascular microbiota can help understand the pathoetiology of the vascular diseases in more detail. On the other, the information gathered through this process can increase the success of vascular transplantation.

In reviewing current reports, we have not found any example that would describe a possible method for identifying healthy human vascular wall tissue microbiota or results characterizing the composition of the human blood vessel microbiota. The aim of our study was to develop a hands-on method for analysing the human vascular wall tissue microbiota and to characterize its healthy composition.

## Methods

In our prospective survey study fourteen human femoral arteries, harvested from fourteen different donors in multi-organ donations between October 2019 and February 2021 in Hungary, were examined to identify the healthy human vascular microbiota.

### Donor characteristics

The median age of the donors was 49.5 (IQR:10) years. Male/female rate of donors was 11/3. The median body mass index (BMI) was 27.8 (IQR:7.9) kg/m^2^. The leading cause of death was cerebral haemorrhage (7 donors; 50%). All essential characteristics of the donors are presented in **Table 1**.

**Table 1:** Donor characteristics.

Characteristic		n = 14
Cause of death	trauma	5 (35.71)
	cerebral ischaemia	2 (14.28)
	cerebral haemorrhage	7 (50)
Age (year)		49.5 (IQR:10)
Female sex		3 (21.42)
BMI (kg/m2)		27.8 (IQR:7.9)
Past medical history	Hypertension	3 (21.42)
	Diabetes	1 (7.14)
	Smoking	7 (50)
Blood type	A	5 (35.71)
	B	2 (14.28)
	AB	1 (7.14)
	O	6 (42.85)
	Rh+	12 (85.71)
	Rh-	2 (14.28)

Data are presented as number of donors (%) or median (IQR). n, number of donors; BMI, body mass index.

### Inclusion and exclusion criteria of allografts

Donor inclusion criteria of the study were based on the national multi-organ donation criteria and rules (Hungarian National Blood Transfusion service., 2020). Briefly, the selection criteria for donors are based on an analysis of the risks associated with the use of tissues. Signs of these risks must be identified through a medical examination, medical history, lifestyle, biological examination, post-mortem examination and any other suitable examination. Donors should be excluded from donating if any of the following criteria apply:The cause of death is unknown., Unknown disease in medical history., Malignant disease., Transmissible spongiform encephalopathies., Any untreated infection at the time of donation., HIV, Any autoimmune disease that may affect the transplanted organ., Any intervention that may modify the results of the donor’s blood tests., Any potencial transmitted disease., Poisoning., The donor was a former recipient., Vaccination with live virus. The allografts from donors above 65 years, with malignancy or with positive bacterial-fungal culture or virus serology tests and the allografts with negative evaluation of the vascular surgeon, who performed the explantation (e.g. significantly injured or calcified allograft), were excluded.

### Ethical considerations, data collection

The study was in full compliance with the principles of the national multi-organ donation and the applicable international and national laws. The anonymous data of the donors were collected prospectively from the electronic health information system of the donation according to the General Data Protection Regulation of the European Union. The study was approved by the institutional review board, by Semmelweis University Regional and Institutional Committee of Science and Research Ethics (approval number: 257/2018).

### Harvesting of allografts and preparing of samples

Femoral arteries were harvested in Budapest from brain-dead donors in multi-organ donations organized by the Organ Coordination Office of the Hungarian National Blood Transfusion Service. All donors had negative serology test of human immunodeficiency virus, hepatitis B virus, hepatitis C virus, syphilis, active Epstein-Barr virus and active cytomegalovirus. The explantation of the femoral arteries (common and superficial femoral artery) was performed under surgical asepsis and sterile techniques. The suitability of the grafts was evaluated by the vascular surgeon, who performed the harvesting. Immediately after explantation, the allografts were placed into a triple sterile plastic bag (Set of Transplantation Bags – sterile 80 00 61H, Raguse GmbH, Ascheberg, Germany) in 500 mL transport solution (Sodium Chloride 0.9% “Baxter” Intravenous Infusion in Viaflo, Baxter Hungary, Budapest, Hungary) containing 4 mg/mL cefazolin (Sandoz GmbH, Kundl, Austria) and 0.4 mg/mL fluconazole (Fresenius Kabi Hungary, Budapest, Hungary) at 4°C. They were transferred in an organ transport box (IGLBox Organ Transporter, Institut Georges Lopez, Lissieu, France) at 4°C to the site of storage and were stored for 12 h at 4°C.

The samples for microbiota analysis were prepared within 24 h after the explantation under sterile conditions in a clean room classified “A” with a background classified “B” used laminar air flow system (European Commission, 2022). From each femoral artery three 3 mm^3^ samples were cut. The samples were placed into a sterile plastic tube (VWR Low Temperature Freezer Vials, VWR International, LLC, Radnor, PA, USA) in physiological saline solution (sodium chloride 0.9% “Baxter” Intravenous Infusion in Viaflo, Baxter Hungary, Budapest, Hungary) and transported immediately for microbiota analysis at 4°C.

### DNA isolation, 16S rRNA gene library preparation and MiSeq sequencing

DNA isolation was performed by ZymoBIOMICS DNA Miniprep Kit (Zymo Research Corp., Irvine, CA, USA) according to the manufacturer’s instructions, after enzymatic dissolution with ProtK (56°C, 3 h). Isolated DNA samples were placed at −80°C until polymerase chain reaction (PCR) amplification. Concentration of genomic DNA was measured using a Qubit2.0 Fluorometer with Qubit dsDNA HS Assay Kit (Thermo Fisher Scientific, Waltham, MA, USA). Bacterial DNA was amplified with tagged primers covering the V3-V4 region of the bacterial 16S rRNA gene. PCR and DNA purifications were performed according to Illumina’s protocol. PCR product libraries were assessed using DNA 1000 Kit with Agilent 2100 Bioanalyzer (Agilent Technologies, Waldbronn, Germany). Equimolar concentrations of libraries were pooled and sequenced on an Illumina MiSeq platform (Illumina, San Diego, CA, USA) using MiSeq Reagent Kit v3 (600 cycles PE).

**Figure 1:**
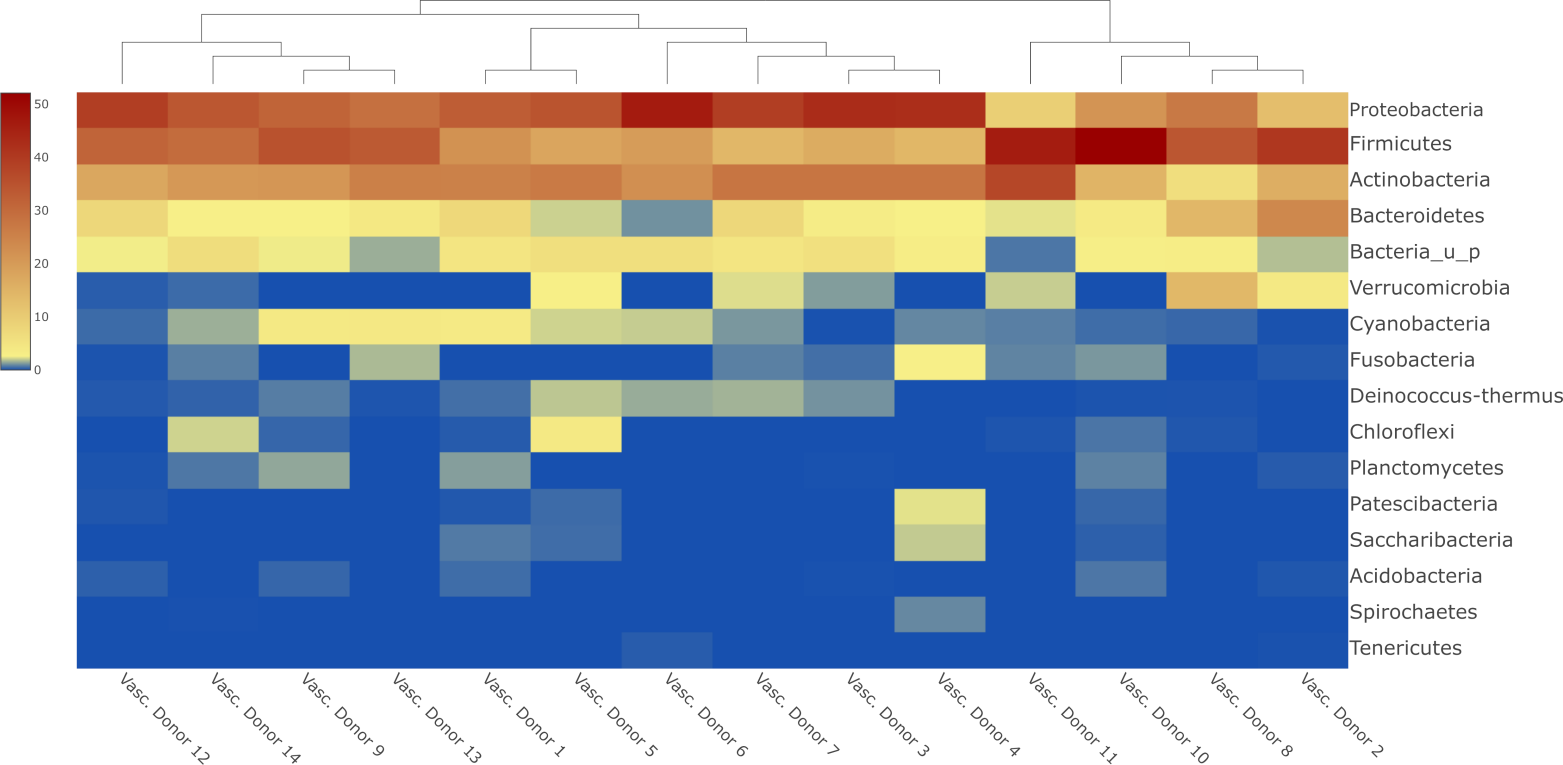
Heatmap about the most abundant phyla in human vascular samples with sample clustering dendogram.

**Figure 2:**
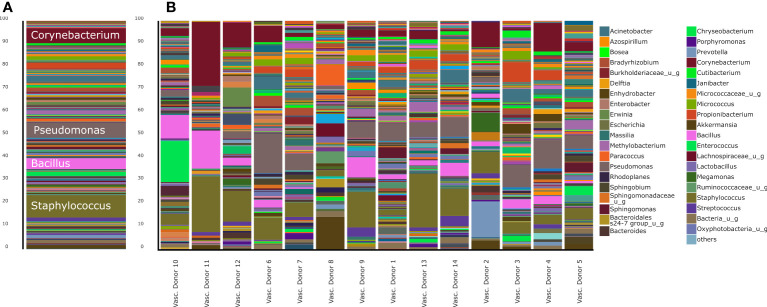
Stacked bar representation of human vascular microbiome composition at genus level. **(A)**, aggregated results and **(B)**, results of separated samples.

**Figure 3:**
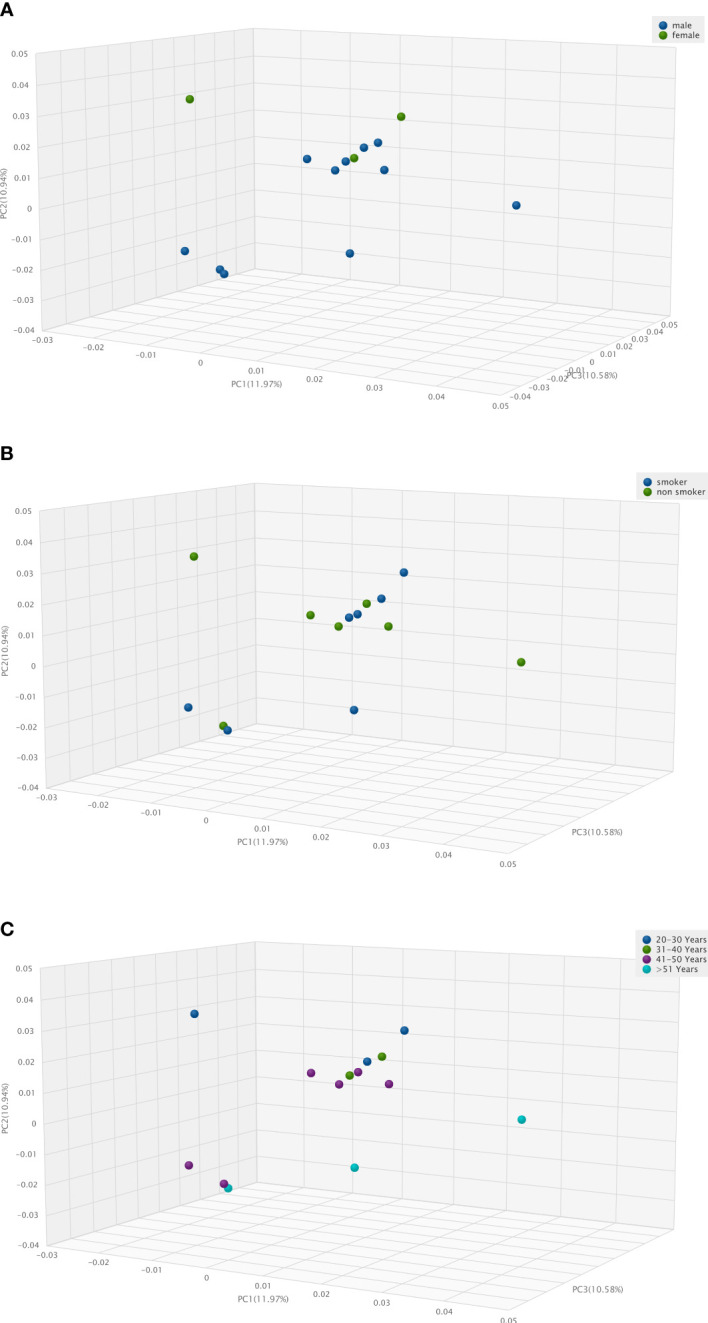
Three-Dimension Principal Component Analysis (3D PCA) of human vascular microbiome. **(A)**, in male and female patients, **(B)**, in smoker and non-smoker patients, **(C)**, according to age distribution.

**Figure 4:**
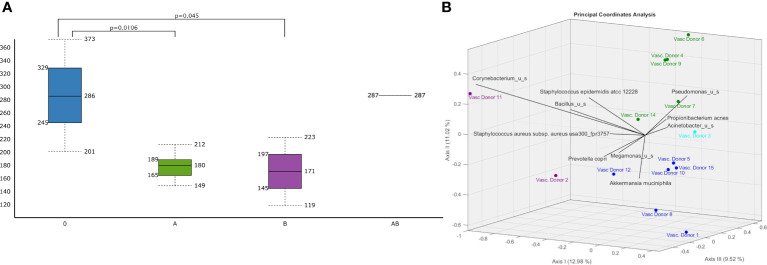
Differences in vascular microbiomic diversity according to A (green)/B(purple)/AB(light blue)/O(dark blue) blood groups. **(A)** Chao1 Alpha Diversity of human vascular microbiome significant differences are indicated with p values and **(B)** bi-plot Jaccard Beta Diversity Principal Coordinate Analysis according to A/B/AB/O blood groups..

**Figure 5:**
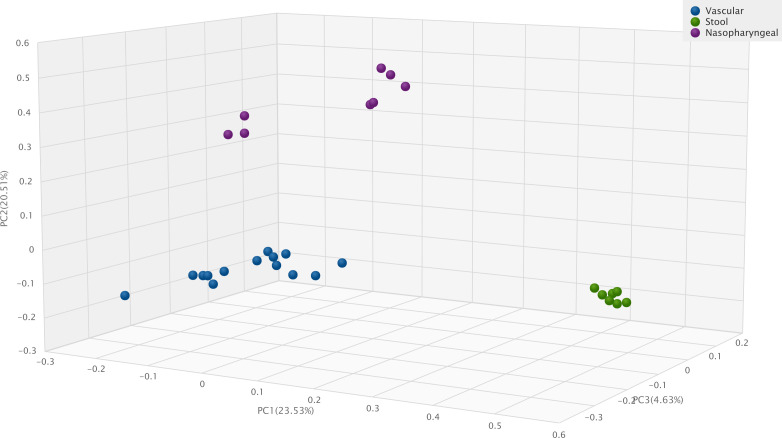
Jaccard Beta Diversity Principal Coordinate Analysis of human vascular, human stool and human nasopharyngeal samples.

In order to evaluate the contribution of extraneous DNA from reagents, extraction negative controls and PCR negative controls were included in every run. To ensure reproducibility, each vascular tissue sample was independently extracted. To avoid false results (e.g.: contamination) and to increase the reliability of the study, all analysis procedures were done in triplicate from 3 different samples of each donor. Raw sequencing data were retrieved from the Illumina BaseSpace and the data were analysed by the CosmosID bioinformatics platform (CosmosID Metagenomics Cloud, app.cosmosid.com, CosmosID Inc. Rockville, MD, USA, www.cosmosid.com) described elsewhere (Yan et al., 2019). Briefly: The raw reads from paired-end fastq files were processed through read trimming (DADA2) to remove adapters as well as reads and bases of low quality. The forward and reverse overlapping pairs were joined together; and with the supplementing unjoined R1 and R2 reads were than used as input for OTU picking. OTUs are identified against the CosmosID curated 16S database using a closed-reference OTU picker and 97% sequence similarity through the QIIME framework. The final results contain taxonomic names, OTU ids, frequency, and relative abundance. Of the 3 samples from each donor, the sample with highest read quantity were selected for comparative studies. **Figures 1**–**5** were generated from the phyla or genus-level filtered abundance score matrices from the CosmosID taxonomic analysis using the CosmosID-HUB bioinformatics pipeline (app.cosmosid.com/comparative). Software for multivariate data analysis of Fathom Toolbox for MATLAB was used to create the Jaccard PCoA biplot diagram of **Figure 4B** (Jones, 2017).

### Statistical analysis

Categorical variables are described as counts of cases and percentages. Continuous variables are described as median with interquartile range. Statistical significance between cohorts of vascular samples were implemented Wilcoxon Rank Sum test for Chao1 Alpha diversity and PERMANOVA analysis for Jaccard PCoA Beta diversity using the statistical analysis support application of CosmosID bioinformatics platform.

We compared the results of current vascular samples with the DNA amounts of faecal and pharyngeal samples from our previous studies (Szabo et al., 2021), obtained using the same DNA isolation and 16S rRNA method. The levels of statistical significance for the differences between the amounts of isolated DNA or amounts of amplified and tagged PCR products – not normally distributed variables – measured in the vascular samples or other frequently tested samples was calculated by the Mann-Whitney U test.

A value of p<0.05 was considered significant.

## Results

From each common-superficial femoral artery samples three smaller pieces were processed separately, for a total of forty-two tested samples from fourteen patients. The median of isolated DNA from vascular samples was 27.8 ng/µL (IQR: 21.4 ng/µL). From this starting amount, which also contains human DNA, after 16S rRNA PCR a median DNA amount of 1.546 ng/µL (IQR: 0.762 ng/µL), after indexing PCR a median DNA amount of 3.947 ng/µL (IQR: 1.996) was amplified. The average length of index PCR products was 667 bp (SD: 55 bp). From the simultaneously processed transport buffers of samples, as from negative controls neither DNA isolation nor 16S rRNA PCR resulted in measurable amounts of DNA.

A total of 5.8 million valid sequences were obtained, resulting in 3.9 million high-quality reads; the median number of reads within one sample was 79485 (IQR: 24511).

The most abundant phyla in the human vascular microbiota were *Proteobacteria* (31.78%), *Firmicutes* (29.18%) and *Actinobacteria* (23.05%) (**Figure 1**). The samples are clustered into three major groups according to the rate of the three most relevant phyla. At the genus level, the most abundant taxa in the human vascular microbiota were *Staphylococcus, Pseudomonas, Corynebacterium, Bacillus, Acinetobacter* and *Propionibacterium* (**Figure 2A**). However, the stacked bar graph clearly shows that the individual samples contained very different proportions of these genera (**Figure 2B**). The median of the Chao1 alpha diversity of genera was 217.5 (IQR: 107).

Of the bacterial taxa that have an indirect effect on the development of atherosclerosis, we found *Porphyromonas gingivalis* in two samples (0.54%, 0.16%), *Prevotella nigrescens* in one sample (0.1%) and all samples contained different amounts of *Enterobacteriaceae spp* with a median of 2.25% (IQR: 1.46). Of the bacteria that are more common in the intestinal flora of healthy people than in patients with atherosclerosis, *Roseburia* was found in 8 samples (median: 0.13%, IQR: 0.08) and *Ruminococcus* occurred in 13 samples (median: 3.07%, IQR: 1.78). *Helicobacter pylori* and *Chlamydophila pneumoniae*, bacteria that can play a direct role in development of atherosclerosis, were not detected in any of the samples.

Due to the low number of cases, we grouped donors based on only a few individual traits and examined differences in microbiota composition. Three-Dimension Principal Component Analysis (3D PCoA) pictures fails to show any significant difference i) between the vascular microbiota of male and female patients **(** **Figure 3A** **)**, ii) in smoker and non-smoker patients (**Figure 3B**), iii) according to age distribution **(** **Figure 3C** **)**.

There were significant differences in the Chao1 alpha diversity of microbiota between the O and A blood groups (p=0.0106) and between the O and B blood groups (p=0.045) (**Figure 4A**) using the Wilcoxon Rank Sum test. Jaccard distance measure was used for assessing the beta-diversities of the microbiota and Principal Coordinate Analysis (PCoA) biplot for visualizing microbiotas **(** **Figure 4B** **)**. There were significant differences using PERMANOVA analysis in the Jaccard beta-diversity values of the microbiotas between O and A blood groups (p= 0.003), between O and B blood groups (p=0.037) and between A and B blood groups (p=0.045).

Compared to previous results of our study group (Szabo et al., 2021), **Figure 5** shows significant differences with Jaccard Beta Diversity PCoA between human vascular and human stool (p=0.001) or human nasopharyngeal (p=0.001) samples.

## Discussion

In the case of visceral tissues previously considered sterile, it has been repeatedly shown that they contain microbial genetic material (Aagaard et al, 2014; Ghaemi et al., 2021; Sookoian and Pirola, 2021). Indeed, hereby we could detect these from healthy arterial wall. We managed to develop a usable protocol for the identification of healthy vascular microbiota by which we were able to characterize the composition of the human blood vessel microbiota.

It is impossible to collect an artery wall sample from a living, healthy person for research purposes, so no one has previously characterized the microbiome composition of a healthy blood vessel wall. Access to this special, unique test material was only possible by processing a small piece of a blood vessel section removed from a dead person intended for vascular allograft transplantation.

Our vascular samples contain less microbial DNA than samples containing physiological bacterial flora. Using the same isolation and sequencing methods the difference is significant - in contrast with our previous results (Szabo et al., 2021) - after 16S rRNA PCR the median bacterial DNA amount is 1.546 ng/µL from vascular samples and 17,4 ng/µL in stool samples (p= 0,002). However, there is no significant difference between the average length of index PCR bacterial products 667 bp in vascular samples and 662 bp in stool samples (Szabo et al., 2021). The microbiota of the human vessel forms a well-defined cluster that is significantly distinct from the microbiota of other human samples. The composition of the vascular microbiota is uniquely and significantly different from either nasopharyngeal or faecal microbiota samples.

Recent accounts report on the blood microbiota of healthy donors with *Proteobacteria*, *Actinobacteria, Firmicutes* and *Bacteroidetes* phyla as the largest representatives (Paisse et al., 2016; Castillo et al., 2019). Three of them, *Proteobacteria, Firmicutes*, and *Actinobacteria* phyla were also identified by us as the most abundant phyla in the human vascular microbiota. Based on these studies, we can assume that the two microbiotas are related to each other. However, their exact composition could be different. While the main phylum of the blood was *Proteobacteria*, over 80% (Paisse et al., 2016), the main phyla of the arterial wall were *Proteobacteria, Firmicutes*, and *Actinobacteria* with almost similar proportions. The result, that there can be a difference between the microbiome of the blood vessel wall and the microbiome of the circulating blood is not surprising. Numerous studies have proven that faecal microbiota only partially reflected the gut mucosal-associated microbiome (Flemer et al., 2017; Yang et al., 2020), and the urine microbiome also differs from the bladder tissue microbiome (Mansour et al., 2020). Based on these examples, we can assume that bacteria transiently present in the blood are also added to the blood microbiome composition, but only a part of them can be detected in the vessel wall. Furthermore, the results of blood microbiome tests can be very different even for patients who consider themselves to be healthy, the presence or absence of e.g. periodontitis or other disorders determines the quantity and quality of viable bacteria in the blood (Damgaard et al., 2015; Damgaard et al., 2021). The vessels we examined did not contain any viable bacteria, because samples with positive culture results were excluded from the study. All of these can result in differences in blood and blood vessel wall microbiome results. In addition to the microbes circulating in the blood, those microbes whose DNA content can be detected in the vessel wall can have a direct effect on the function or pathological changes of the vessel wall with their metabolic products or antigens derived from them.

Numerous studies have found association between cardiovascular diseases (coronary, cerebrovascular or peripheral) and specific bacteria in different sites of body (Blanchard et al., 1993; Kozarov et al., 2005; Caula et al., 2014; Ahmed and Tanwir, 2015; Clifford and Hoffman, 2015; Jie et al., 2017; Liu et al., 2019; Verhaar et al., 2020; Jin et al., 2021). Increased amounts of *Porphyromonas gingivalis*, *Tannerella forsythia*, *Prevotella intermedia*, *Aggregatibacter actinomycetemcomitans, Treponema denticola, Prevotella nigrescens, Fusobacterium nucleatum, Eikenella corrodens* and *Parvimonas micra* in the oral cavity may play a role in endovascular pathogenic processes by enhancing systemic inflammatory parameters (Caula et al., 2014; Tapashetti et al., 2014; Ahmed and Tanwir, 2015; Damgaard et al., 2017). Only two of our healthy vascular samples contained *P. gingivalis* and only one sample contained *P. nigrescens* from the bacteria mentioned above. Gut microbiota can also impact the cardiovascular physiology with the produced metabolites that enter the blood (Verhaar et al., 2020). A causal effect of Trimethylamine-N-Oxide (TMAO) on atherosclerosis has been established (Eshghjoo et al., 2021), and the TMAO producer members of *Enterobacteriaceae* family have higher abundance in the gut microbiota of patients with symptomatic atherosclerosis compared to healthy controls (Jie et al., 2017). Nevertheless, Short Chain Fatty Acid (SCFA) - producing bacteria from the *Eubacterium, Roseburia*, and *Ruminococcaceae* families have higher abundance in the gut microbiota of healthy patients than in atherosclerosis ones’ (Liu et al., 2019). Each of our samples contained different low amounts of bacterial DNA from members of the *Enterobacteriaceae* family, and the occurrence of *Roseburia* and *Ruminococcaceae* species in the samples was also common in small amounts. Assuming that bacteria may enter the circulation from the intestinal tract of the patients, the DNA detected from the vascular wall is an indirect information that the intestinal flora of the patient may contain short fatty acid-producing strains. The bacteria that have been proven to play indirect role in the pathological changes of the vessel wall were not detectable, or only in minimal amounts, in the healthy vessel wall microbiome.

The most important bacteria whose DNA have been found in the atherosclerotic plaque and whose direct role in the development of atheromas has been demonstrated are *P. gingivalis, A. actinomycetemcomitans, C. pneumoniae* and *H. pylori (* Kozarov et al., 2005; Rosenfeld and Campbell, 2011; Clifford and Hoffman, 2015 *)*. Of these bacteria, only *P. gingivalis* occurred in 2 cases in low amounts in our healthy human vessel wall microbiota samples.

In our study, the donors were divided into well-separated vascular microbiota clusters according to ABO blood group system. Mäkivuokko and Gampa reported similar results for gut microbiota (Makivuokko et al., 2012; Gampa et al., 2017). However, Davenport et al. found no association between ABO antigen status and gut microbiota composition in a large cohort of 1500 twins (Davenport et al., 2016). In a genome-wide association study in 8956 German individuals an indirect influence of ABO histo-blood groups on gut microbiome was also identified (Ruhlemann et al., 2021). In a large mosaic pig population, the deletion in the gene encoding N-acetyl-galactosaminyl-transferase was associated with lower abundance of *Erysipelotrichaceae-* bacteria that can import and catabolize N-acetyl-galactosamine *-*in gut microbiome. Since alpha 1-3-N-acetylgalactosaminyltransferase and alpha 1-3-galactosyltransferase are the genes determining the ABO blood group in humans, a molecular explanation proves the connection between the gut microbiome composition and the blood groups (Yang et al., 2022). There is already molecular-genetic proof of the relationship between the gut microbiome and ABO blood groups, but the recently detected blood vessel wall microbiome - blood group correlation can only be confirmed after examining a larger number of samples.

The composition of the intestinal and additional microbiotas influences the success of organ transplantations. Certain bacteria could precipitate allograft rejection by producing metabolites that activate host cell-mediated and humoral immunity (Mitchell, 2019; Baghai Arassi et al., 2020; Campbell et al., 2020; Dery et al., 2020). Vascular allograft transplantation has a high number of graft-related complications such as extended calcification or aneurysmal degeneration, the exact cause of which is unknown despite extensive research of the last decades (Chakfe et al., 2020). However, as the role of the microbiota has arisen in case of a series of organ-graft rejection, it could be also one to induce vascular transplantation-failures. Further studies are needed to determine whether antigens or metabolites of bacteria in the blood vessel wall affect the outcomes of vascular allograft transplantation.

The limitations of this study could be the low number of the donors, however the number of multi-organ donations are limited and there is no other ethical way for harvesting healthy vascular samples. The concept of “healthy” is a difficult one to define. However, multi-organ donations have very strict criteria, which can ensure the structural and functional health of the harvested organs despite possible comorbidities. Nonetheless, future studies investigating subgroups of donors with different comorbidities could be useful.

Summarizing the results of our research the human arterial wall has a unique microbiota that is significantly different in composition from that of other areas of the human body. The most important genera were *Staphylococcus, Pseudomonas, Corynebacterium, Bacillus, Acinetobacter* and *Propionibacterium*, which were found in variable but dominant quantities in all vessel wall samples. In addition, the donors were divided into well-separated vascular microbiota clusters based on ABO blood group system. Follow-up studies with a larger number of samples are needed to reveal the relationship between the microbiome composition of the vessel wall and the success of transplantation. Our present study is the first methodological description that can be used to examine the microbiome of the vessel wall. The pathological role of a microbe in the damaged vessel wall can only be proven if we know the microbiome associated with the healthy vessel wall. Our present study provides a basis for following research that investigates the direct role of the microbiota in vascular wall disorders and the success of allograft transplantations.

## Data availability statement

The datasets presented in this study can be found in online repositories. The names of the repository/repositories and accession number(s) can be found below: https://www.ncbi.nlm.nih.gov/, PRJNA748211, PRJNA224116.

## Ethics statement

The studies involving human participants were reviewed and approved by Semmelweis University Regional and Institutional Committee of Science and Research Ethics (approval number: 257/2018). Written informed consent for participation was not required for this study in accordance with the national legislation and the institutional requirements. 

## Author contributions

Conceptualization: PS, DS, EO, LH, Data curation: GK, LH, EO, Funding acquisition: PS, LH, DS, Investigation: NM, KP, EO, LH, Methodology: PS, LH, EO, NM, KP, Project administration: LH, NM, EO, Resources: EO, DS, LH, JJ, Software: EO, DS, JJ, Supervision: PS, EO, DS, Validation: PS, LH, EO, DS, Visualization: EO, DS, JJ, Writing - original draft: EO, LH, Writing – review and editing: PS, DS, EO, JJ. All authors contributed to the article and approved the submitted version.

## Funding

This work was supported by Semmelweis University-Eötvös Lóránd Research Network, Human Microbiota Study Group No”0272” and by the Supplementary Research Fellowship for Excellence of Semmelweis University (EFOP-3.6.3-VEKOP-16-2017-00009).

## Conflict of interest

The authors declare that the research was conducted in the absence of any commercial or financial relationships that could be construed as a potential conflict of interest.

## Publisher’s note

All claims expressed in this article are solely those of the authors and do not necessarily represent those of their affiliated organizations, or those of the publisher, the editors and the reviewers. Any product that may be evaluated in this article, or claim that may be made by its manufacturer, is not guaranteed or endorsed by the publisher.
